# Audiovestibular Symptoms in Systemic Autoimmune Diseases

**DOI:** 10.1155/2018/5798103

**Published:** 2018-08-19

**Authors:** Massimo Ralli, Vittorio D'Aguanno, Arianna Di Stadio, Armando De Virgilio, Adelchi Croce, Lucia Longo, Antonio Greco, Marco de Vincentiis

**Affiliations:** ^1^Department Oral and Maxillofacial Sciences, Sapienza University of Rome, Rome, Italy; ^2^Department of Sense Organs, Sapienza University of Rome, Rome, Italy; ^3^San Camillo Hospital IRCCS, Venice, Italy; ^4^Humanitas Clinical and Research Center, Via Alessandro Manzoni 56 20089 Rozzano, Italy; ^5^Department of Otolaryngology, University G. D'Annunzio of Chieti-Pescara, Chieti, Italy

## Abstract

Immune-mediated inner ear disease can be primary, when the autoimmune response is against the inner ear, or secondary. The latter is characterized by the involvement of the ear in the presence of systemic autoimmune conditions. Sensorineural hearing loss is the most common audiovestibular symptom associated with systemic autoimmune diseases, although conductive hearing impairment may also be present. Hearing loss may present in a sudden, slowly, rapidly progressive or fluctuating form, and is mostly bilateral and asymmetric. Hearing loss shows a good response to corticosteroid therapy that may lead to near-complete hearing restoration. Vestibular symptoms, tinnitus, and aural fullness can be found in patients with systemic autoimmune diseases; they often mimic primary inner ear disorders such as Menière's disease and mainly affect both ears simultaneously. Awareness of inner ear involvement in systemic autoimmune diseases is essential for the good response shown to appropriate treatment. However, it is often misdiagnosed due to variable clinical presentation, limited knowledge, sparse evidence, and lack of specific diagnostic tests. The aim of this review is to analyse available evidence, often only reported in the form of case reports due to the rarity of some of these conditions, of the different clinical presentations of audiological and vestibular symptoms in systemic autoimmune diseases.

## 1. Introduction

The inner ear has been considered for a long time an immune-privileged site, spared from organ-specific autoimmunity and rarely involved in systemic autoimmune diseases thanks to the blood-labyrinthine barrier [1]. Lehnhardt [2] was the first to hypothesize that sudden or rapidly progressive sensorineural hearing loss (SNHL) could be the result of an autoimmune process against the inner ear. McCabe [3] showed the success of steroid and cytotoxic treatment in a cohort of patients with bilateral progressive SNHL, suggesting an autoimmune pathogenesis. Recently, several studies showed inflammatory cells in the inner ear, describing the presence of resident cochlear macrophages in animal models and the recruitment of inflammatory macrophages to the cochlea [4]. In 2016, O'Malley et al. identified cells with staining characteristics and morphology consistent with macrophages/microglia in the human cochlea [4]; the presence of these cells in patients with autoimmune diseases suggests that they may have an important role in inner ear pathology due to the increased level of proinflammatory cytokines and reactive oxygen species (ROS) induced by microglia [4].

There is growing interest for inner ear involvement in systemic autoimmune diseases [5, 6]; this condition should be considered in patients with audiovestibular dysfunction presenting a constellation of symptoms consistent with systemic autoimmunity or with a preexisting diagnosis of autoimmune disease [7, 8]. Inner ear involvement in systemic autoimmune diseases should be distinguished from primary autoimmune inner ear disease, a condition in which the immune response acts directly against the inner ear [6, 7].

Inner ear involvement in autoimmune diseases is estimated to account for less than 1% of all cases of acquired hearing loss [7] and follows gender and demographic characteristics of autoimmune disorders, with higher prevalence in female patients between their thirties and fifties [5].

A correct identification of inner ear involvement in patients with systemic autoimmune diseases is essential for the possibility of near-complete hearing restoration with appropriate treatment [9]; however, it is often misdiagnosed due to variable clinical presentation, limited knowledge, sparse evidence, and lack of specific diagnostic tests.

The aim of this review is to analyse the available evidence of the different clinical presentations of audiological and vestibular symptoms in systemic autoimmune diseases, although this is often only reported in the form of case reports due to the rarity of some of these conditions.

## 2. Inner Ear Involvement in Autoimmune Diseases

### 2.1. Pathophysiology of Inner Ear Involvement in Autoimmune Diseases

Pathophysiology of inner ear involvement in systemic autoimmune diseases is still unclear and may be related to circulating antibodies against a number of inner ear antigens leading to antibody-dependent cell-mediated cytotoxicity, the activation of the complement system, a direct action of cytotoxic T cells, or immune complex-mediated damage [5, 7, 8, 10–14].

The immune complex deposition seems to play a central role in inner ear involvement; it leads to vasculitis of inner ear vessels that determines atrophy of the stria vascularis and SNHL. The deposition of immune complexes reduces the calibre of the auditory arteries with a consequent decrease in blood flow; blood flow reduction induces an oxygen deficit that increases the ROS level responsible for damage to hair cells and spiral ganglion [15–17]. This pathogenic mechanism appears to be the major factor involved in cochlear and vestibular damage in systemic autoimmune diseases, especially when affecting the labyrinthine artery, the common trunk of the inner ear vascularization system [18].

Temporal bone studies clearly explain the mechanism of damage. The vascular ischemia, underlying vasculitis, initially determines the atrophy of the stria vascularis and hair cell death; the progression of the inflammation initiates bone inflammatory processes such as necrosis or cochlear fibrosis [8], more evident in the final stages of the disease [19].

### 2.2. Audiovestibular Symptoms in Systemic Autoimmune Diseases

Audiovestibular dysfunction in systemic autoimmune diseases may have different clinical presentations with high interindividual variability [20]. Hearing loss is the most common condition, followed by tinnitus and vertigo [8].

Characteristics of hearing impairment are extremely variable. The hearing loss is typically sensorineural, affecting mainly high frequencies [8, 9], although low-frequency and mid-frequency hearing loss are common in cases of vasculitis [20]. The general pattern of SNHL is rapidly progressive over a period of weeks to months, with great timing variability among different systemic diseases [21]; fluctuations in hearing are common, although the overall course is a progressive deterioration of auditory function [9]. Hearing loss is mainly bilateral and asymmetric; however, cases of unilateral SNHL that manifests in the contralateral ear after a variable time have been described [21]. In some cases, a temporary and acute blood flow reduction in the inner ear may be related with the onset of sudden sensorineural hearing loss (SSNHL), with complete or partial recovery after restoration of normal perfusion. SSNHL is common in patients with inner ear involvement following systemic autoimmune diseases, and may be the presenting symptom in some cases [5, 20]. Despite hearing loss being mainly sensorineural, autoimmune diseases can also induce a conductive hearing loss (CHL). In these cases, CHL may follow the effusions of the middle ear and the inflammation of Eustachian tube mucosa or involvement of the ossicular chain [22–24].

Tinnitus in systemic autoimmune diseases is mainly found in association to hearing loss. It has been established that the decrease of peripheral input following hearing loss can trigger neuroplastic reactions up to the auditory cortex responsible for the onset of tinnitus. Therefore, it is probable that peripheral auditory dysfunction could initiate central changes that eventually lead to tinnitus onset in patients with autoimmune diseases [25–28].

Vestibular symptoms, such as rotational vertigo or disequilibrium, may follow temporary occlusion of the labyrinthine or the anterior vestibular artery [1, 11], and they often mimic primary inner ear disorders such as Menière's disease [3, 21].

A list of systemic autoimmune diseases that have been reported to be associated to audiovestibular symptoms, along with relevant literature references, is shown in Table 1.

#### 2.2.1. Systemic Lupus Erythematosus

Systemic lupus erythematosus (SLE) is an autoimmune disease with multiorgan involvement and an incidence higher in women (82%–96%) than in men (4%–18%) [29].

The audiovestibular symptoms that are present in patients with SLE may follow antibody/antigen direct reactions, cytotoxic action, or immune complex deposition [15]. Vasculitis following immune complex deposition is the major cause of cochlear and vestibular damage in SLE patients. Immune complexes deposit in the auditory artery reducing the vessel calibre with a consequent decrease in blood flow and oxygen deficit; this stimulates the release of ROS that damage the hair cells and the spiral ganglions with consequent hearing impairment [16].

SNHL is the most common audiovestibular symptom in SLE, with a prevalence of between 6% and 70% [15]. Hearing loss may be bilateral or unilateral, slowly progressive, or sudden; it mainly affects high frequencies, mimicking the typical presbycusis pattern, but may also involve low and middle frequencies [15]. Maciaszczyk et al. [30] and Roverano et al. [18] described progressive, bilateral SNHL involving high frequencies. Khalidi et al. [31] reported unilateral SNHL involving mid and high frequencies (500, 1000, 2000, and 3000 Hz) associated with a 16% word discrimination score as demonstrated by speech audiometry. Sperling et al. [32] described both bilateral and slowly progressive SNHL and unilateral SSNHL in patients with SLE.

Tinnitus is often associated with hearing loss in SLE, most probably following peripheral deafferentation [33–35].

The vestibular system could be involved in SLE, but vertigo and dizziness have rarely been reported [35, 36]. Balance disorders as a consequence of SLE have been observed also in children [37]; however, the incidence of vestibular symptoms may be underestimated due to their slowly progressive onset and consequent compensation by the somatosensory system and vision.

#### 2.2.2. Cogan's Syndrome

Cogan's syndrome (CS), first described in 1934, is a rare autoimmune disorder characterized by ocular and audiovestibular symptoms [39]. CS develops in young adults, mostly during their first three decades of life [40, 41]. The origin of CS is still unclear. Antibodies against Cogan peptide have been found in serum of patients with CS. Also, this peptide antigen shares sequence homology with CD148 and connexin 26, both involved in congenital deafness [42].

CS includes a large spectrum of clinical manifestations. Haynes et al. [43] defined two types of CS, a typical and an atypical variant. Typical CS is defined by ocular symptoms, classically presenting as nonsyphilitic interstitial keratitis (IK), audiovestibular symptoms similar to those of Menière's disease (recurrent episodes of hearing loss, tinnitus, and vertigo), and an interval between the onsets of ocular and audiovestibular manifestations of less than 2 years. Atypical CS is characterized by different inflammatory ocular manifestations, with or without IK; audiovestibular symptoms (usually progressive hearing loss); and, most important, a delay of more than 2 years between the onset of ocular and audiovestibular manifestations. In many cases, it is difficult to differentiate between the two types of CS because some patients do not present IK at the onset of the disease or, alternatively, they develop this condition during the following years. Systemic manifestations are much more frequent in atypical CS and can be used in the differential diagnosis between the two types [41].

Inner ear involvement in CS has been reported with a prevalence of between 31% and 45% [40–44]. The most common audiovestibular manifestations in CS are hearing loss, vertigo, tinnitus, ataxia, and oscillopsia [41]. These symptoms can appear at any time during the course of the disease [44]. Hearing loss may be both unilateral and bilateral, often presenting as SSNHL with fluctuations or progressive worsening over time. Progression to complete bilateral hearing loss has been reported in almost 50% of patients during the follow-up period, whereas permanent hearing loss in one ear was observed in 20% of patients [44].

Tinnitus usually follows auditory deafferentation [41]. Abnormal vestibular function is found in 90% of patients with CS; at least 20% of the patients present spontaneous or gaze-induced nystagmus. Rarely, patients show clinical symptoms of vestibulopathy that last for days or weeks from the time of onset without spontaneous resolution that frequently results to hospitalization [44].

#### 2.2.3. Sarcoidosis

Sarcoidosis is an inflammatory multisystem disease with unknown origin. CNS involvement is reported in about 5–7% of patients with systemic sarcoidosis, called neurosarcoidosis (NS) [46].

A cranial nerve neuropathy affecting the facial and optic nerves is a common finding in up to 80% of NS patients [46, 47].

Audiovestibular involvement is common in sarcoidosis. In a review of 50 patients with NS [48], a high incidence of audiovestibular manifestations was noted. Hearing loss was present in 49/50, unilateral in 25% and bilateral in 75% of the patients. Tinnitus was reported in 30 patients (61%), and vestibular impairment was recorded in 32/50 (64%) including vertigo, dizziness, and benign paroxysmal positional vertigo. A complete vestibular function testing was performed in 24 patients and found abnormalities in 23 (96%). Of those, six (25%) had unilateral alterations, 16 (67%) had bilateral alterations, and one (8%) had a nonlocalizing dysfunction. In another review, Babin [49] reported SNHL in approximately 90% of reported cases, characterized by sudden or rapidly progressive onset, and vestibular symptoms with abnormal vestibular functioning tests in a similar percentage of cases. In almost 50% of the cases, at least partial hearing recovery was achieved after high-dose systemic steroid administration, while balance disorders recovered either spontaneously or after treatment [49].

A recent study [50] reported two new cases of SSNHL due to probable NS, each having a quite different clinical course. In one case, unilateral SSNHL and facial palsy were the presenting symptoms of NS, while in the second, unilateral SSNHL occurred despite ongoing immunosuppressive treatment for NS.

#### 2.2.4. Rheumatoid Arthritis

Rheumatoid arthritis (RA) is a chronic, inflammatory disease affecting nearly 0.6% of the general population [52]. Principal symptoms referred by patients are articular and periarticular, although RA can involve other organs including the heart, lung, skin, and eye [52].

Several events can lead to audiovestibular alterations during the course of RA; thus, a wide variation in the prevalence of different types of hearing impairment in RA patients may be found [53]. SNHL is the most common type of hearing impairment in RA patients ranging from 25% to 72% [53]. Conductive hearing loss and mixed hearing loss have also been reported, although less frequently [53].

A prospective case-control study [24] compared hearing disturbances in patients with RA with a control group. In 60% of the RA patients, SNHL was observed and the difference was statistically significant at 500 Hz, 1 kHz, and 2 kHz in both ears. CHL was reported in 17.1% of the RA patients compared to 5.7% of patients in the control group, with a statistically significant difference.

Pathogenesis of CHL in RA is still poorly understood; several hypotheses have been proposed. A laxity of the middle ear transducer mechanism [54] was proposed although other authors [24] suggested increased stiffness of the ossicular system.

#### 2.2.5. Antiphospholipid Syndrome

The antiphospholipid syndrome (APS) is an acquired disorder characterized by the presence of antiphospholipid antibodies such as anticardiolipin (aCL) and lupus anticoagulant (LAC) antibodies causing hypercoagulability. The characteristic triad of the disease is the association of specific antibodies, arterial or venous thrombosis, and/or pregnancy morbidity and mortality [55]. APS is associated with microthrombosis, causing cutaneous manifestations like purpuric eruptions, livedo reticularis, and skin ulcerations. The involvement of the retina may cause amaurosis fugax [56].

The involvement of the inner ear has been reported in APS and may be related to antibodies targeting the small vessels of the labyrinthine circulation. Endothelial cells within the cochlear circulation might be activated by antiphospholipid antibodies directly or by inducing the formation of free radicals that, secondarily, damage the endothelium. These upregulated endothelial cells would initiate local microthrombus formation and subsequent ischemia to the target organ [57].

The association of aCL or LAC antibodies and SNHL was firstly reported by Naarendorp and Spiera [56] in six patients with SLE or a lupus-like syndrome. Toubi et al. [58] studied sudden and progressive SNHL in 30 patients showing that in the control group, no one had aCL antibodies, whereas 27% of the patient group had aCL antibodies in low to moderate concentration. In a subsequent study, Toubi et al. [59] reported that 31% of patients with idiopathic SSNHL were positive for aCL, compared with only 6% of matched control subjects. A prospective study [60] had 168 patients with progressive SNHL who underwent a screening panel of blood tests for autoimmune disease including aCL antibodies, anti-B2 glycoprotein, and LAC. In this population, forty-two patients (25%) had at least one elevated antiphospholipid antibody marker and twenty patients had two or more positive test results, suggesting that antiphospholipid antibodies could be involved in the pathogenesis of some forms of inner ear dysfunction, related to a microthrombus formation in the labyrinthine vasculature.

#### 2.2.6. Polyarteritis Nodosa

Polyarteritis nodosa (PAN) is a systemic necrotizing vasculitis that mainly affects medium-sized arteries, although small arteries may also be involved [61]. The incidence of PAN ranges from 0 to 1.6 cases per million, and the prevalence of this disease is approximately 31 cases per million [62]. PAN affects men more frequently than women and occurs in all ethnic groups; the average age at onset is approximately 50 years [63].

The pathogenesis of idiopathic PAN remains enigmatic, although the clinical response to immunosuppressive therapy suggests that immunological mechanisms play an active pathogenic role. As in other forms of vasculitis, the presence of impaired endothelial function could reflect direct endothelial cell activation and damage resulting from primary inflammatory vasculitis or proinflammatory cytokines or antibodies [62].

Clinical manifestations of PAN include nonspecific constitutional manifestations, such as sickness, weight loss, fever, arthralgia, and myalgia. Dysfunction or damage of target organs may produce specific symptoms, often caused by occlusion or rupture of inflamed arteries. The most frequently involved territories are the skin and peripheral nervous system [62].

SNHL is often reported in PAN [64–66] and, in rare instances, may occur as the presenting symptom of the disease [67, 68]. Hearing loss is typically bilateral and symmetrical, with sudden onset [67, 69] or a rapidly progressive course [65, 70]. Alterations found in auditory brainstem responses (ABR) suggest an inner ear involvement, and the involvement of low frequencies may resemble those found in endolymphatic hydrops [69]. Tinnitus, vertigo, and occasional nausea and vomiting may also be found in patients with PAN [67].

#### 2.2.7. Behcet's Disease

Behcet's disease (BD) is a chronic systemic relapsing syndrome affecting young adults and characterized by the presence of recurrent oral and genital ulcers, ocular inflammation, and skin lesions caused by a vasculitis involving small vessels [72].

Hearing loss is a common compliant in BD, it is mainly bilateral and predominantly affects high frequencies; several studies have reported SNHL following cochlear impairment ranging from 12% to 80% in BD patients [72, 73]. No relationship has been found between age or disease duration and inner ear involvement [73, 74]. A recent survey [75] of 65 BD patients reported that audiovestibular complaints were found in 47% of patients. The most common symptoms were tinnitus (11%), hearing loss (10%), and vertigo (8%); a case of unilateral SSNHL was also reported.

Nearly half of BD patients report orthostatic disequilibrium [72]. Studies of the vestibular function [73, 74] showed that BD mainly causes peripheral lesion rather than damage to the central vestibular tracts, although another study [76] showed a higher prevalence of central vestibular syndrome in BD patients. Magnetic resonance studies did not show any degenerative conditions of basal ganglia and brainstem atrophies in BD patients with abnormal vestibular function tests [75, 76]. Neural involvement in BD (neuro-Behcet) may appear with dizziness or vertigo as initial symptoms, mimicking a vestibular neuritis [77].

#### 2.2.8. Takayasu's Arteritis

Takayasu's arteritis (TA), also known as aortitis syndrome, is a vasculitis that mainly affects large elastic arteries with symptoms caused by organ ischemia, aneurysm formation, and inflammation. TA is more prevalent in women of reproductive age, and clinical features usually reflect limb or organ ischemia that follow gradual stenosis of the involved arteries [78, 79].

The aetiology of hearing impairment in TA remains unknown [79]; it has been hypothesized that hearing loss follows the elevation of serum immune complexes that deposits in the inner ear or reversible circulatory disturbances with hypercoagulability in response to arterial disease [80]. Although TA involves medium and large calibre arteries, Noel et al. reported that the occlusion of small retinal vessels is a possible microcirculatory complication in TA; common immunopathology mechanisms with hearing loss could be hypothesized [81]. Moreover, Maruyoshi et al. speculated that hearing loss in TA could have a vascular background based on reversible circulatory disturbances due to vasculitis and/or some autoimmune pathogenesis in the inner ear, involving especially hair cells [80].

Only a few cases in the literature reported an association of TA with SNHL [82–84]. Hearing loss is often progressive, although it can be stable or fluctuating; is usually bilateral and asymmetric; develops over several weeks to months; and mainly involves high frequencies. SNHL may also present as a SSNHL. A good response to corticosteroid therapy has been reported for SNHL in TA, although it may also persist despite therapy [80]. Recently, Ralli et al. described a case of a 36-year-old woman with TA who had two episodes of SSNHL involving one ear at a time with an 11-month delay between each episode of treatment with hyperbaric oxygen therapy associated to corticosteroids, with significant improvements in both ears [85, 86].

#### 2.2.9. Relapsing Polychondritis

Relapsing polychondritis (RP) is a rare connective tissue disorder affecting organs containing collagen, such as the eye, cartilage tissue, and skin. The diagnosis is based on clinical features and no specific test for this disease is available; thus, definitive diagnosis takes a long time, and often, the prognosis is poor [87]. Recurrent bouts of inflammation may lead to a permanent destruction of involved structures such as cartilage of the ears, nose, larynx, tracheobronchial tree, and cardiovascular system [88]. McAdam et al. [89] proposed diagnostic criteria for RP when three or more of the six clinical features are present: recurrent chondritis of both auricles; nonerosive, seronegative inflammatory polyarthritis; chondritis of the nasal cartilages; ocular inflammation (conjunctivitis, keratitis, scleritis, and uveitis); respiratory tract chondritis affecting laryngeal and tracheal cartilages; and cochlear and/or vestibular dysfunction (SNHL, tinnitus, and vertigo).

Auricular chondritis is a quite specific sign of RP. It is present in 20% of patients at the onset of the disease and in 90% during the course of the disease [90]. One or both ears can be affected. The ear concha is swollen, red, or less often purplish, hot, and painful even at the slightest contact. The ear lobe, which does not contain the cartilage, is spared.

Audiovestibular impairment is reported in 40–54% of all patients with RP [91]. These changes can represent the initial symptoms heralding the outbreak of the disease or appear after the onset of other symptoms. Typical manifestations can be bilateral or unilateral, are usually of sudden onset, and appear as perceptive deafness or tinnitus combined with or without vertigo and nausea [89]. CHL can be a result of a serous otitis following chondritis of the eustachian tube [23]. Further, the SNHL in RP patients has been suggested to be the result of inflammation of the internal auditory artery or its cochlear branch [92] or due to autoantibodies against the cochlea and vestibular organ [91]. This could explain the near-complete hearing recovery in patients after treatment with corticosteroids.

#### 2.2.10. Wegener's Granulomatosis

Wegener's granulomatosis (WG) is an autoimmune disease of unknown aetiology characterized by necrotizing granulomatous inflammation of the respiratory tract, necrotizing glomerulonephritis, and systemic vasculitis that affects predominantly small vessels.

Although the pulmonary, nasal, and renal manifestations of WG are well described, hearing symptoms are less appreciated. Studies available in the literature report a prevalence of audiovestibular symptoms in 8% to 65% of patients with WG, mainly auditory symptoms with SNHL [94, 95] or CHL in cases of WG involving the middle ear [96]. Audiometric patterns of WG have been described as typically flat, although sometimes additional high frequency losses may coexist and differential diagnosis with noise exposure or age-related hearing loss may be difficult [96]. Hearing impairment may also present as SSNHL; Bakthavachalam et al. [97] identified that hearing loss was present in 56% of patients suffering from WG and SSNHL was the most common form, occurring in 47% of cases.

Hearing loss may also be a presenting symptom of WG. SNHL in WG is believed to be largely irreversible, therefore potentially adding to the patient's cumulative disability [96]. SNHL evaluation and monitoring are therefore recommended for appropriate patient management and could suggest a worsening of disease that may address to a specific treatment like cyclophosphamide rather than either methotrexate or azathioprine [94, 95].

#### 2.2.11. Susac's Syndrome

Susac's syndrome (SS) is a rare immune disease characterized by encephalopathy, branch retinal artery occlusion, and SNHL [98]. SS has a higher prevalence in females, with a male: female ratio of 1 : 3.5 [99].

The pathophysiology of SS is not entirely clear. Antiendothelial cell antibodies (AECAs) in SS have been recently documented [99]. Potential targeted antigens suggested in studies focusing on AECAs include cytoskeletal proteins (*β*-actin, *α*-tubulin, and vimentin), glycolytic enzymes (glucose-3-phosphate-dehydrogenase and α-enolase), and the prolyl-4-hydroxylase *β* subunit, a member of the disulfide isomerase family [100].

Clinical manifestations of SS are thought to be caused by autoimmune-mediated occlusions of microvessels in the brain, retina, and inner ear that lead to a characteristic clinical trial of central nervous system (CNS) dysfunction [101]. At clinical onset, the most common manifestations are CNS symptoms, observed in two-thirds of patients, followed by visual symptoms and hearing disturbances. Unfortunately, only a small number of patients (around 13%) show the characteristic symptoms of SS at disease onset; thus, definitive diagnosis is often delayed [101].

Hearing loss can be a dramatic and severely debilitating feature of SS; it may be mild and insidious or may be fluctuating, mimicking Menière's disease [102]. A loss of low or middle frequencies is typical, suggesting a vulnerability of the cochlear apex to microinfarction; loss of high frequencies can also occur [99]. Hearing loss often occurs overnight and may affect both ears. Dörr et al. refer to this clinical behaviour as the “bang-bang hearing loss” [101]. Severe hearing loss is often accompanied by vertigo and a roaring tinnitus; the occlusion of cochlear and vestibular arterioles may be the cause of these symptoms [99]. In these patients that often develop severe/profound SNHL and can no longer benefit from hearing aid amplification, cochlear implantation is a valid therapeutic option.

#### 2.2.12. Sjögren's Syndrome

Primary Sjögren's syndrome (pSS) is a chronic autoimmune disease characterized by xerostomia and xerophthalmia due to lymphocyte infiltration of both salivary and lacrimal glands. It may also occur as a systemic disease involving the kidneys, lungs, liver, vessels, and lymph nodes. pSS mainly affects women in the fourth-fifth decade of life, and presenting symptoms are often oral and ocular [104]. In this context, autoantibodies to cardiolipin and M3 muscarinic receptors (mAchRs) in the serum of pSS patients are suspected to play a pathogenic role in the onset of progressive hearing loss and neurological complications [104].

Audiovestibular involvement in patients with pSS has been reported in the literature with a prevalence ranging from 22% to 46%. Boki et al. [105] reported in a pSS patient the presence of SNHL affecting preferentially high frequencies. Tumiati et al. [106] reported SNHL in 46% (14/30) of patients with pSS. Ziavra et al. [107] diagnosed SNHL in 22.5% (9/40) of pSS patients. Hearing loss as presenting complaint in pSS is quite uncommon and only limited to case reports [22]; SSNHL was recently reported in a 62-year-old female treated with high-dose methylprednisolone (250 mg) infusion for 5 days with successful hearing restoration [108].

The high prevalence of cranial neuropathies is a known condition in pSS, mainly with trigeminal and facial nerve involvement [109].

Although pSS patients tend to have a higher prevalence of SNHL compared to the general population, no evidence of damage to the central auditory pathways was reported [106]. However, the prevalence of audiovestibular symptoms in pSS might be underestimated, suggesting that their association with pSS was not previously made because it had not been actively sought [106].

#### 2.2.13. Myasthenia Gravis

Myasthenia gravis (MG) is the most common autoimmune disorder affecting the neuromuscular junction, characterized by muscle weakness and fatigue [111]. Weakness, the typical clinical symptom of MG, affects facial, ocular, bulbar, respiratory, or limb muscles and worsens after muscular activity [112].

In healthy subjects, acetylcholine (ACh), the primary neurotransmitter of the efferent auditory system, has been found to enhance the electromotility of outer hair cells (OHC) binding to acetylcholine receptors (AChRs), which are localized on the postsynaptic membrane of OHC [113].

In patients with MG, autoantibodies against AChRs were reported to bind with AChRs on OHCs, inducing a progressive loss of AChRs that decreases OHC electromotility [114]. This cascade of events induces apoptosis in all three rows of OHCs, evolving into a clinically evident SNHL in rare cases [115]. The efferent auditory system has been investigated using contralateral acoustic stimulation (CAS) [116]. CAS produces physiological suppression of otoacoustic emissions [117] protecting the hair cells from noise of moderate to high intensity [118]. A reduced CAS effect has been reported in patients with MG compared to control subjects, suggesting a possible role of the progressive reduction of beta subunits of nicotinic AChRs associated to the destruction of the basal membrane and OHCs due to prolonged exposure to autoantibodies [114, 116].

At MG onset, patients do not refer hearing loss and pure tone audiometry (PTA) is often within normal range. A specific test studying, the activity of the OHC, otoacoustic emissions (OAE), may show some abnormalities, as OAE have been found to exhibit greater sensitivity to incipient cochlear damage compared to PTA, particularly for high frequencies [115, 119]. A study on 16 MG patients reported a clinical hearing loss in 30% of the patients, while 100% of the patients exhibited abnormal distortion product otoacoustic emissions (DPOAEs) and transient evoked otoacoustic emissions (TEOAEs) [114]. Therefore, OAE should always be performed in MG patients because they can early detect the MG-related effects on the ACh-innervated auditory system [111].

Additional audiological symptoms, such as tinnitus, should be always considered and investigated although seldom reported [27].

Vertigo, also reported in patients with MG, seems to be more related to musculoskeletal alterations rather than to vestibular impairment [112].

#### 2.2.14. Multiple Sclerosis

Multiple sclerosis (MS) is traditionally considered an autoimmune inflammatory demyelinating disease of the central nervous system (CNS). The autoimmune pathogenesis of MS is still debated; recently, it has been hypothesized that it may be a homogeneous degenerative process analogous to primary neurodegenerative diseases [121]. As an exacerbating and remitting immune-mediated disorder of interfascicular oligodendrocyte-produced myelin, MS can impair acutely and transiently any CNS neural system, including the auditory pathways [122].

The evidence of a clear presence of macrophages in the human temporal bone of patients affected by autoimmune diseases [15] support the hypothesis that in MS, the autoimmunity mechanisms also affect the structures of the inner ear; hair cells and auditory and vestibular spiral ganglion neurons may be subject to the attack of lymphocytes, and their damage may present with SNHL and vertigo. The microglia, a cell population that belongs to the macrophage family and that is normally represented in the brain, has been shown to be active in aggressive forms of MS (phenotype M1). Temporal bone studies could suggest that microglia can migrate to the internal auditory canal and to the cochlea [4]. M1 microglia can demyelinate cochlear and vestibular structures causing SSNHL or vertigo; such episodes may be temporary due to the relapsing-remitting phases of MS that activates and inactivates the M1 microglia.

In the literature, several reports showed MS-related hearing deficits. Hearing loss may occur when MS involves both the peripheral and the brainstem auditory pathways [123]; however, in some case, MS lesions involving the auditory pathways may not determine a clinically evident hearing impairment [124]. In rare cases, SSNHL may be the only presenting symptom of MS and may appear early in the course of the disease with good prognosis and little or no residual hearing deficit [125].

MS patients typically report a difficulty in speech perception, especially in noise [126]. This alteration is due to an abnormal auditory processing, such as problems with dichotic listening tasks and auditory temporal processing [126]. Performance of chronic MS subjects in speech reception threshold (SRT) is normal in the standard clinical level (70 dB above the SRT); however, when lower levels are used, performance significantly decreases compared to age-matched controls suggesting a deficit in cognitive processing, such as attention and auditory discrimination, which is especially required in binaural integration of sound [127].

Furthermore, studies have shown that 40% to 55% of individuals with MS have at least an episode of dysarthria or speech alteration characterized by slowness, slurring, or difficulties in production or comprehension [127].

Disequilibrium in MS is often related to internuclear ophthalmoplegia, and multiple nystagmus constitutes the most typical vestibular signs of MS, although peripheral equilibrium may coexist [128]. Multidirectional nystagmus without latency may be an atypical central sign, and differential diagnosis with peripheral disorder, such as benign paroxysmal positional vertigo, can be more difficult, although adaptation and fatigue of nystagmus play a central role in differential diagnosis. When the clinical findings are not clear and ex-adjuvantibus criteria cannot be adopted, vestibular-evoked myogenic potential (VEMP) may be proposed for the differential diagnosis of positional vertigo in association with careful clinical history and otoneurologic examination [128].

Audiovestibular symptoms in young, neurologically normal subjects, especially when spontaneous recovery occurs, could represent an early sign of MS even when no demyelinated plaques are visible in the central nervous system; it would be recommended to evaluate these subjects with clinical, radiological, and electrophysiological tests to exclude peripheral incipient MS [129].

#### 2.2.15. Other Autoimmune Conditions

Other autoimmune diseases associated to audiovestibular symptoms include Hashimoto's thyroiditis, mixed cryoglobulinemia, giant cell arteritis (GCA), Vogt-Koyanagi-Harada's disease, and Ulcerative Colitis.

Thyroid autoimmunity seems to affect the inner ear, particularly inducing hearing loss at lower frequencies [131].

In mixed cryoglobulinemia, unilateral SNHL has been found in 22% of patients following immune complex deposit in labyrinthine vessels determining both audiological and vestibular symptoms [132].

Hearing loss has been reported with a prevalence ranging from 7% to 100% in several case series of patients with GCA, a multisystemic vasculitis mainly involving large- and medium-sized blood vessels [133]. In a series of 44 patients with GCA, PTA at the time of diagnosis showed auditory dysfunction in all patients [134]. In some patients, hearing loss was progressive and appeared as an initial manifestation [135].

Bilateral rapidly progressive SNHL and tinnitus and vestibular manifestations have been observed in 48% to 62% of patients with Vogt-Koyanagi-Harada's disease [136].

In a retrospective study, SNHL was found in about 2% of patients with ulcerative colitis [137]. In these patients, OAE play a central role as they may indicate a cochlear involvement even when normal hearing thresholds are present [138].

### 2.3. Audiovestibular Diagnostic Workup in Systemic Autoimmune Diseases

The audiovestibular symptoms in systemic autoimmune diseases are often related to the entity of the autoimmune damage, as they may follow an inflammatory process in the inner ear or a direct macrophage aggression of the inner and—mainly—outer hair cells [15].

The current literature agrees that SNHL is the most common auditory symptom of systemic autoimmune diseases [5], but due to the different presentation forms (sudden or progressive) and severity (mild to severe) of SNHL, an early correlation between the symptom and the systemic autoimmune disease may be difficult. Furthermore, audiovestibular symptoms found in autoimmune conditions are also common to other conditions such as diabetes and hypertension. For these reasons, a correct differential diagnosis of the cause of the audiovestibular involvement is of utmost importance.

The diagnostic process in patients presenting with a variety of audiovestibular symptoms should begin with individual medical history and family history followed by traditional audiovestibular tests. The most important audiological test battery in all cases should include PTA possibly extended to the high-frequency region, TEOAE and DPOAE, and ABR [5, 129]. Vestibular testing should include a basic vestibular exam integrated with caloric test, video head impulse test, and VEMPs [129]. The aforementioned test batteries should be performed at the onset of the audiovestibular symptom and during follow-up to monitor the course of the disease. Audiovestibular examination should be integrated, when an autoimmune condition is suspected, with specific blood tests as summarized in Table 2 [139].

### 2.4. Treatment Approaches to Audiovestibular Symptoms in Systemic Autoimmune Diseases

The treatment of audiovestibular symptoms should first aim at preserving the function, such as hearing preservation and/or restoration in patients with SNHL, and then at solving disability, distress, and quality of life. If an underlying autoimmune disease is suspected, treatment should be started after complete blood exams; in fact, steroid therapy, that is, commonly used as first-line treatment for SSNHL and other audiovestibular symptoms, may have an effect on the underlying autoimmune systemic disease and delays its diagnosis.

In patients with a diagnosis of systemic autoimmune disease, the treatment of the audiovestibular symptoms is usually strictly related to that of the systemic condition. Common treatment options for systemic autoimmune diseases that may present an audiovestibular involvement are summarized in Table 3.

The most common treatment for SSNHL is systemic or intratympanic administration of high doses of corticosteroids, associated with hyperbaric oxygen treatment in cases of SLE, APS, and TA [85, 86]. Other associated treatments include antioxidant compounds to avoid progression of SNHL [142], hearing aids to support the residual hearing function, or cochlear implants in case of severe and profound SNHL [143, 144].

Tinnitus is commonly treated with approaches aimed to restore hearing function, with antidepressant drugs when a psychological involvement is detected, and using oral supplements that combine antioxidants and vasoactive substances [145].

Vertigo can be treated with high doses of corticosteroids [146] associated to betahistine, a strong antagonist of the histamine H3 receptor and a weak agonist of the histamine H1 receptor, that improves vascularization of the inner ear [145]. Additional therapeutic approaches include metoclopramide and antidepressant drugs (inhibitor of D1 receptor) that act on central function by reducing the sensation of vertigo, nausea, and gastrointestinal symptoms [146]. For chronic dizziness, specific rehabilitation treatments can be used to favor central vestibular compensation and restore normal balance function [147].

## 3. Conclusion

Audiovestibular symptoms may be found in a variety of autoimmune diseases, and diagnosis is essential to increase the chances of restoration when specific therapy is promptly initiated. Inner ear involvement in autoimmune diseases is ascertained by the history, clinical findings, an immunologic evaluation of the patient's serum, and response to immunosuppressive therapies, following exclusion of other known causes.

Audiovestibular symptoms could play a role in the diagnostic process of autoimmune diseases as they may be an early-onset symptom—and in some cases, the only symptom—of an autoimmune condition. Furthermore, they may be useful to monitor the progression of the systemic disease.

Systemic autoimmune diseases should always be considered in patients with audiovestibular symptoms such as progressive/fluctuating SNHL with no other explainable cause. When a systemic autoimmune disease involving the inner ear is suspected, predisposing factors must be investigated, such as noise exposure, ototoxic treatments, previous ear surgery, trauma, meningitis, or family history of hearing loss. The exclusion of concomitant conditions may be challenging, especially in the case of presbycusis- or noise-induced hearing loss. The low prevalence of these conditions, the heterogeneity of studies available in literature, and the absence of randomized trials are the factors that limit the knowledge of inner ear involvement in systemic autoimmune diseases along with underestimation of the problem and consequent undertreatment.

## Figures and Tables

**Table 1:** Systemic autoimmune diseases associated to audiovestibular symptoms.

Autoimmune disease	Prevalence of audiovestibular involvement	Classification	Literature reference
Systemic lupus erythematosus	6–70%	Systemic autoimmune rheumatic disorders	[15, 16, 18]; [29–37]; [38]
Cogan syndrome	31–45%	Systemic vasculitis	[39–44]; [45]
Sarcoidosis	5–96%	Systemic granulomatous diseases	[46–50]; [51]
Rheumatoid arthritis	25–72%	Systemic autoimmune rheumatic disorders	[24]; [52–54]
Antiphospholipid syndrome	Case reports only	Autoimmune hypercoagulable condition	[55–60]
Polyarteritis nodosa	Case reports only	Systemic vasculitis	[61–70]; [71]
Behcet's disease	12–80%	Systemic vasculitis	[72–77]; [45]
Takayasu's arteritis	Case reports only	Systemic vasculitis	[78–85]; [79]; [86]
Relapsing polychondritis	40–54%	Autoimmune connective tissue disorder	[23]; [87–92]; [93]
Wegener's granulomatosis	8–65%	Systemic vasculitis	[94–97]
Susac's syndrome	Case reports only	Systemic vasculitis	[98–102]; [103]
Sjögren's syndrome	22–46%	Systemic autoimmune rheumatic disorders	[22]; [104–109]; [110]
Myasthenia gravis	22–34%	Autoimmune condition affecting neuromuscular junction	[111–119]; [120]
Multiple sclerosis	1–28%	Autoimmune inflammatory demyelinating disease	[4, 15]; [121–129]; [130]
Hashimoto thyroiditis	Case reports only	Autoimmune thyroid disease	[131]
Mixed cryoglobulinemia	22%	Systemic vasculitis	[132]
Giant cell arteritis	7–100%	Systemic vasculitis	[133–135]
Vogt-Koyanagi-Harada's disease	48–62%	Systemic granulomatous diseases	[136]
Ulcerative colitis	2–5%	Autoimmune inflammatory bowel disease	[137, 138]

Summary of systemic autoimmune diseases that have been reported to be associated to audiovestibular symptoms, along with reported prevalence of audiovestibular involvement, classification, and relevant references.

**Table 2:** Blood tests commonly used in patients with audiovestibular symptoms suggestive for a systemic autoimmune condition.

Test	Classification
Red and white cell counts	General blood test
Coagulation test (aPTT, PT)	General blood test
Creatine kinase (CK)	General blood test
Alanine transaminase (ALT)	General blood test
Aspartate aminotransferase (AST)	General blood test
Erythrocyte sedimentation rate (ESR)	Inflammatory markers
C-reactive protein (CRP)	Inflammatory markers
Ferritin	Inflammatory markers
Enzyme-linked immunosorbent assay (ELISA)	Immunologic analysis
Rheumatoid Factor (RF)	Antibody
Anti-cyclic citrullinated peptide antibody (CCP)	Antibody
Anti-nuclear antibody (ANA)	Antibody
Anti-double-stranded DNA (anti-dsDNA)	Antibody
Antiextractable nuclear antigen (anti-ENA)	Antibody
Antisignal recognition particle (anti-SRP)	Antibody
Anti-Mi2	Antibody
Antineutrophil cytoplasmic antibody (ANCA)	Antibody
Lupus anticoagulant (LAC)	Antibody
Antiphospholipid autoantibodies (aPL)	Antibody
Anticardiolipin (aCL)	Antibody
Complement (C3, C4, and B)	Complement
Cryoglobulins	Immunoglobulin

Summary of most relevant blood tests used to investigate a possible autoimmune condition.

**Table 3:** Common treatment for systemic autoimmune diseases with audiovestibular involvement.

Disease	Treatment	Reference
Systemic lupus erythematosus (SLE)	SLE without major organ manifestations: antimalarials and/or glucocorticoids; nonsteroidal anti-inflammatory drugs may be used judiciously for limited periods of time in patients at low risk for drug-induced complications; in nonresponsive patients, immunosuppressive agents such as azathioprine, mycophenolate mofetil, and methotrexate should also be considered	[38]
Cogan's syndrome (CS)	Prednisone 1 mg/kg/day for two weeks and then tapered over 3 to 6 months; methotrexate for long-term treatment; alternative treatments are cyclophosphamide, azathioprine, tacrolimus, and rituximab	[45]
Sarcoidosis	High dose of corticosteroids (20–40 mg/daily) for 6 to 18 months; high-dose intravenous n-methyl-prednisone with doses of up to 30 mg/kg for 1–5 days has been commonly recommended for treatment of refractory neurosarcoidosis; in addition, methotrexate, azathioprine, and TNF-alpha antagonists	[51]
Rheumatoid arthritis (RA)	Methotrexate at disease onset (10–15 mg/week) and then 20 mg/week for 4–8 weeks; it is possible to use prednisolone at high dosage (40–60 mg) and tapering to 7.5 mg at week 6 for a total of 12 weeks	[140]
Antiphospholipid syndrome (APS)	Chronic treatment with low dose of acetylsalicylic acid	[57]
Polyarteritis nodosa (PAN)	PAN without viral syndrome: prednisone 1 mg/kg/day and then tapering when remission is reached	[71]
Behcet's disease (BD)	Steroid treatment with azathioprine; for resistant cases, azathioprine + interferon + TNF-*α* antagonists	[45]
Takayasu's arteritis (TA)	Prednisone 1 mg/kg/day; additionally, it is possible to use immunosuppressants such as methotrexate, azathioprine, mycophenolate mofetil, leflunomide, tacrolimus, and TNF-alpha antagonists	[79]
Relapsing polychondritis (RP)	Corticosteroid treatment at high dosages; in addition, colchicine, methotrexate, azathioprine, intravenous immunoglobulins, minocycline, and leflunomide	[93]
Wegener granulomatosis (WG)	Prednisone or equivalent 1 mg/kg/day, sometimes preceded in severe cases by intravenous methylprednisolone pulses (7.5–15 mg/kg/day) for 1–3 consecutive days; after two weeks, tapering with a decrease of 10% every two weeks for a total of 6 months; in case of long-term treatment (>2 years), 5 mg/day; is also possible to use cyclophosphamide and rituximab for maintenance therapy	[141]
Susac syndrome (SS)	High-dosage corticosteroids; additionally, intravenous immunoglobulin, plasma exchange azathioprine, mycophenolate mofetil, methotrexate, cytochrome P450 enzymes, and cyclosporine A	[103]
Sjögren's syndrome (pSS)	Cyclosporine A for local treatment of eye disease; colchicine and steroid treatment are used; controversial use of rituximab	[110]
Myasthenia gravis (MG)	Immunosuppressant therapy; in addition, treatment with insulin,thyroid hormones, and pyridostigmine	[120]
Multiple sclerosis (MS)	Immunomodulating therapy: T cell suppressor (alemtuzumab, daclizumab); B-cell modulators (rituximab, ocrelizumab); unique anti-inflammatory agents (laquinimod); hormones (estriol); 3-hydroxy-3-methylglutaryl-coenzyme A reductase inhibitors; vitamin D	[130]

Treatment options for systemic autoimmune conditions, along with relevant references.
